# Patient information and emotional needs across the hip osteoarthritis continuum: a qualitative study

**DOI:** 10.1186/s12913-016-1342-5

**Published:** 2016-03-12

**Authors:** Espen Andreas Brembo, Heidi Kapstad, Tom Eide, Lukas Månsson, Sandra Van Dulmen, Hilde Eide

**Affiliations:** Faculty of Health Sciences, University College of Southeast Norway, Papirbredden - Drammen kunnskapspark. Grønland 58, 3045 Drammen, Norway; Department of Behavioural Sciences in Medicine, Faculty of Medicine, University of Oslo, Oslo, Norway; Department of Emergency and Orthopaedics, Vestre Viken HF, Drammen Hospital, Dronninggata 28, 3004 Drammen, Norway; NIVEL (Netherlands Institute for Health Services Research), Otterstraat 118-124, 3513 CR Utrecht, The Netherlands; Department of Primary and Community Care, Radboud University Nijmegen Medical Centre, Geert Grooteplein Noord 21, 6500 HB Nijmegen, The Netherlands

**Keywords:** Osteoarthritis, Needs assessment, Professional-patient relations, Decision-making

## Abstract

**Background:**

Osteoarthritis (OA) is the most common form of arthritis worldwide, affecting a growing number of people in the ageing populations. Currently, it affects about 50 % of all people over 65 years of age. There are no disease-modifying treatments for OA; hence preference-sensitive treatment options include symptom reduction, self-management and surgical joint replacement for suitable individuals. People have both ethical and legal rights to be informed about treatment choices and to actively participate in decision-making. Individuals have different needs; they differ in their ability to understand and make use of the provided information and to sustain behaviour change-dependent treatments over time.

**Methods:**

As a part of a larger research project that aims to develop and test a web-based support tool for patients with hip OA, this paper is a qualitative in-depth study to investigate patients’ need for information and their personal emotional needs. We invited 13 patients to participate in individual interviews, which were audiotaped. The audio-tapes were transcribed verbatim and analysed using an inductive thematic analysis approach.

**Results:**

The thematic analysis revealed a pattern of patients’ information and emotional needs, captured in several key questions relevant to the different stages of the disease experience. Based on these results and research literature, we developed a model illustrating the patients' disease experience and treatment continuum. Six phases with accompanying key questions were identified, displaying how patients information and emotional needs arise and change in line with the progression of the disease experience, the clinical encounters and the decision-making process. We also identified and included in the model an alternative route that bypasses the surgical treatment option.

**Conclusion:**

Patients with hip OA are in great need of information both at the time of diagnosis and further throughout the disease development and care continuum. Lack of information may result in unnecessary and dysfunctional misconceptions, underuse of potentially helpful treatment options and uninformed decisions. Patients need continuous support from health professionals and their families in order to find and consider effective treatment strategies.

## Background

Osteoarthritis (OA) is the most common form of arthritis and a leading cause of disability among older adults [1]. While OA is uncommon in people under the age of 45 years, nearly 60 % of all people over the age of 75 are affected by it. In an American prevalence study of hip OA 36 % reported hip symptoms, 28 % had radiographic hip OA, 10 % had symptomatic hip OA, and 2.5 % had moderate/severe radiographic hip OA [2]. The prevalence of symptomatic hip OA in the Norwegian population is reported to be 5.5 % [3]. Overall, women over 50 years of age face up to 50 % higher risk of OA than men. This gender difference, however, is less significant in hip OA than in knee OA [4].

OA is characterized by pain and stiffness, causing subsequent loss of functional capability and independence, with limitations in daily living activities and impaired quality of life [5, 6]. As a result, OA is described as affecting the patient’s sense of self and well-being, particularly because of changes in their ability to participate in valued roles and relationships [7]. Given that there is currently no cure for OA, the main goals of treatment are to relieve pain and to preserve physical function. Conservative management strategies typically include education, advice, exercise, weight control, walking aid provision, physiotherapy, activity modification, and pain medication [8]. A Dutch study showed that hip OA patients were under the care of general practitioners for seven years, on average, before they were referred to orthopaedic evaluation for hip replacement. This demonstrates that there is a significant time frame in which to apply and optimize conservative treatment options [9].

Patients’ with hip OA experience their condition as painful, chronic, and incurable but susceptible to control through one or more of the aspects of treatment recommended by their health care provider. The more serious and symptomatic the patients experience the condition, the less positive they feel about the management strategies used to control it [10]. Exercise therapy is a central component of the treatment pathway for OA and has strong evidence [11], but adherence problems are evident [12, 13]. The aim of exercise therapy is to improve muscle strength and joint mobility, often requiring considerable patient commitment over long periods of time.

Turner and colleagues explored the beliefs about the causes of OA among primary care patients [14]. Patients believed that OA and painful joints are an inevitable aspect of ageing rather than an indication of disease. The patients struggled constantly to make minimal use of drugs and maximum use of other management strategies such as resting and avoiding activities. The reluctance to use drugs was prompted by fears of dependency, side effects, and effectiveness waning with long-term use. What worries patients about exercise as treatment, and negatively affects adherence, is the belief that exercise increases the wear and tear of the joint. This indicates the urgent need for information in an early phase of OA. One other study found that adherence to pain medication differed from adherence to other prescribed medications. Perceptions of and attitudes toward pain played an integral role in participants’ adherence. In general, participants minimized their pain and claimed to have a high pain tolerance. This made them reluctant to take pain medication and when they did so, they took a lower dose of medication or took it less frequently than prescribed [15]. Alami and colleagues [16] identified the views of patients and care providers regarding the management of knee OA. The participants felt that their GPs did not take their complaints seriously. They also felt that practitioners are technically focused, paying more attention to the knee than to the individual, and they feel that not enough time is being spent on education and counselling. The care providers emphasized the difficulty in elaborating treatment strategies and the need for a tool to help in decision-making. This may also be the case for persons with hip OA. According to the NICE guidelines, patients who are refractory to conservative treatment and experience symptoms that have a substantial impact on their quality of life should be referred for consideration of hip replacement surgery [17]. Total hip replacement (THR) is a well-established, cost-effective surgical procedure with overall excellent short and long-term results [18, 19], and declining complication rates [20]. Norwegian surgeons performed about 6320 total hip replacements for OA patients in 2014 [21]. Patients undergoing THR experience reduced pain levels and improved function and quality of life [22], and most patients are satisfied [23, 24]. A recent report from the Nordic Arthroplasty Register Association demonstrates that 86 of 100 patients have a functional replaced hip joint without the need for revision after 15 years [25].

The optimal timing for surgery is not known [26, 27], but may be more important than previously acknowledged. Patients with poor pre-operative function are more likely to have postoperative pain and low postoperative physical function [28, 29]. In a prospective study of 165 patients undergoing hip or knee replacement, Fortin and colleagues postulate that performing surgery earlier in the course of functional decline leads to better outcomes in terms of pain and physical function [30]. Because many of the patients with hip OA are older, they may suffer from multi-morbidity and pain in multiple joints. It can be argued that THR is an acute intervention in the context of a chronic disease, and that decision-making should take into account these experiences as a whole, in the context of patient’ preferences, needs and values [31]. With this perspective, it is evident that patient care would benefit from insight into patients’ needs.

### Aim of the study

To our knowledge, there are no studies exploring patents’ emotional and informational needs across the whole care continuum, the studies focussed either on the process related to surgical decision-making or to early phases and self-management support. In the Norwegian Health coordination reform, *Proper treatment – at the right place and right time* [32], there is a strong emphasis on how to strengthen and safeguard the individual patients’ role within the health system. The system is currently undergoing a process of comprehensive change to become better coordinated, to meet the needs of a changing population, and to guarantee patients equal access to good, equitable and balanced health and care services. In this process, successful implementation of structures and systems for more cohesive care pathways is regarded as one important step. This pathway approach is used because it is regarded as helpful in orienting all involved systems and services toward assisting the individual to cope with life or to restore functioning [32]. This is also in line with the recent political ambition in Norway to enhance the person-centeredness of the system and create “the patient’s health service” [33].

In this qualitative study, we examine, across the disease continuum, the (changing) informational and emotional needs in patients with hip osteoarthritis. Based on these perspectives, we developed a model to describe a typical care pathway based on the patient’s experiences and needs illustrated by introspective questions that reflect patients’ needs at different stages throughout the disease. The key research questions in this study are: 1) What information and emotional needs do patients living with hip OA have? 2) How do patients’ needs change over time as the disease progresses? 3) How does decision-making about total hip replacement take place?

## Methods

### Study design

The study has an explorative qualitative design. The individual semi-structured interview was considered to be the most appropriate method for collecting data so that participants were given an opportunity to speak openly about their personal “hip OA journey”. Interviewing can be defined as a ‘professional conversation’, having the objective of getting a participant to talk about their experiences and perspectives and to capture their language and concepts in relation to a topic that the interviewer has determined [34, 35].

### Sample

A purposive sample of patients with hip OA were included from two settings; the hospital setting prior to scheduled surgery and the GP setting. Seven participants were recruited from an orthopaedic outpatient clinic at a local hospital in the South-eastern part of Norway. The letter of invitation and consent form for participation were sent together with the notice of a scheduled surgical appointment. The second group of six participants was recruited from a general practitioner’s office situated at a remote municipality in the Northern part of Norway. A take-home letter of invitation was given to eligible patients by their general practitioners during a planned consultation. Having read the information, interested participants were asked to contact the researcher to schedule an interview. The inclusion criteria were that the participants had a confirmed diagnosis of primary hip OA, were able to communicate in Norwegian and were cognitively capable of participating in an individual in-depth interview of approximately one hour in duration. Furthermore, participants of both genders and different age groups were included. The recruitment procedures at both settings continued until data saturation was considered reached; the point at which new data stopped generating any substantial new ideas [34].

### Data collection

The research questions were explored using individual interviews with a sample of patients who were most likely to be able to share lived experiences that illuminate important insights related to the aim of the study. The first group of participants were individually interviewed in private rooms at the hospital hotel one day prior to elective total hip replacement surgery. It was proposed that these patients could provide valuable perspectives regarding their experiences throughout a relatively long history with hip pain, including how and why they came to the decision to undergo hip surgery. The interviews were planned and performed in the evening to that the doctors and nurses had finished their routine work with admissions. It was discussed with the participants whether it was appropriate to conduct the interviews the day before their elective surgery, and they were offered alternative option, but all informants found the suggested time to be convenient. The second group of informants were interviewed at the local health centre where the GP office was situated. The reason for recruiting these participants was to reach out to persons who manage the disease in a primary care context, and to learn from the experiences they had along the OA treatment and care continuum.

The interviews were audiotaped and conducted in the manner of a conversation, although a semi-structured interview guide was prepared. This explorative approach gave the participants an opportunity to freely disclose and discuss issues that were important to them, rather than respond to specific pre-selected questions. A typical interview was initiated by highlighting essential information about the study and its purpose, and then letting the participant talk freely about his or her “hip journey”, prompted by an open question like “can you start by telling about yourself and how your life is affected by your hip OA?”. Recurrent follow-up questions were *“can you describe in words how your pain feels?”, “what strategies help you cope with your hip pain?”, “what/how have you learned about osteoarthritis?”, “does your pain have any consequences for your social life?”* and *“can you tell me (if) how and why you have decided to undergo hip replacement?”* When the interview was presumed to be finished, the audio-recorder was stopped. The participant was then given some time to settle, whereupon some participants disclosed additional thoughts and perspectives that hadn’t been discussed previously. The audio-recorder was then re-started by the interviewer, but the participant was explicitly made aware of the opportunity and right to delete this part of the interview.

After each interview, the researcher made notes in order to preserve immediate reflections and important features of the conversation. The participants completed a self-reported questionnaire of basic demographic data, duration of OA symptoms, help-seeking behaviour and self-reported comorbidities. This information was collected to acquire relevant data on the characteristics of the participants.

### Research ethics

The study is approved by the Regional Committee for Medical and Health Research Ethics (Ref.: 2013/1231) [36] and complies with the ethical principles stated in the Helsinki declaration [37]. All participants were informed of the goals and design of the study and were assured of confidentiality before they provided written informed consent to participate. At the time of the interview, participants were re-informed and given time to ask any questions they might have.

### Qualitative analysis

Each audio recording was transcribed verbatim in word processing software by the first author, aided by a basic notation system and transcription kit. The transcripts were imported into NVivo (Version 10), a software program that allows systematic coding and retrieval of qualitative data. Inspired by Braun and Clarke, we used an indicative approach to thematic analysis guided by a six-phased analytical process [34, 35]. Braun and Clarke vouch for the rigorous, independent and flexible qualities of this process and explain it as “*a method for identifying, analysing and reporting patterns (themes) within data”* [35]. A theme is defined as *“an abstract entity that brings meaning and identity to a recurrent experience and its variant manifestations. As such, a theme captures and unifies the nature or basis of the experience into a meaningful whole”* [38].

At the initial coding stage, two researchers (EB and TE) independently read and coded two interview transcripts. This collaborative coding process sought to ‘calibrate’ the analytic approach by facilitating structured discussions of possible interpretations. The coded extracts were carefully compared and modified, and preliminary themes were defined. The inductive analytic approach proceeded with iterative and comprehensive coding across the remaining dataset. During this process, each of the coded transcripts was re-checked against the audio-tapes to make sure that the themes bridged the essence of the transcripts with the research questions in focus. When the results had been summarized through major themes, we conducted discussions with nurses and orthopaedic surgeons with the purpose to ascertain their ‘common sense’ opinions of the accounts, based on their experiences from working on a daily basis with hip OA patients. This contributed with a sense of reassurance that the main results and discussions points were realistically presented, as seen from their clinical experience. The first author also spent time observing out-patient consultations and had informal conversations about the research questions and preliminary results with in-patients and their care providers at the ward. The ecological validity of the study was by this strategy strengthened [34].

## Results

### Sample

Nine of the 13 participants had experienced hip symptoms for more than six years, and a subset of five patients for more than ten years. Four of the participants were still working, but were partly on sick leave. Both genders are evenly represented with a wide age distribution, the youngest at 59 years old, and the oldest 88. All seven participants in Group One underwent elective hip replacement the day after the interviews. One participant in group two was accepted for surgery, while two participants were referred and were awaiting orthopaedic evaluation. One participant had previously undergone hip replacement for OA in the contralateral hip. One participant had recently undergone revision surgery due to postoperative femur fracture. A majority had other health problems in addition to their hip OA, such as fibromyalgia, rheumatoid arthritis (RA) and diabetes. Most had access to the Internet and used it at on a regular basis, but only a few used it to seek health information. The interviews had a mean duration of approximately 69 min. All participants in Group One were treated with an anterior total hip replacement technique. The characteristics of the participants are summarized in Table 1.

**Table 1:** Characteristics of participants in Group One (ID 1–7) and Group Two (ID 8–13)

ID	Age	Gender	Marital status	Children	Internet access	Internet usage	Education level	Work status	Time with hip-pain	Self-reported comorbidity	Interview duration (min)
1	85–89	Female	Married	Yes	Yes	Never	Junior high school	Retired	6–7 years	Acute back-pain (recent injury)	133
2	80–84	Female	Widow	Yes	No	N/A	High school	Retired	2–3 years	Diabetes	68
3	60–64	Male	Married	Yes	Yes	Weekly	High school	Mechanic	8–9 years	Hemochromatosis	68
4	70–74	Female	Widow	Yes	Yes	Daily	High school	Retired	6–7 years	RA	65
5	75–79	Male	Married	Yes	Yes	Daily	High school	Retired	4–5 years	Bilateral Hip OA	61
6	70–74	Female	Single	No	Yes	Weekly	High school	Retired	>10 years	RA, shoulder OA	69
7	65–69	Male	Single	No	Yes	Daily	University	Retired	4–5 years	None	80
8	65–69	Female	Married	Yes	Yes	Weekly	University	Retired	4–5 years	Fibromyalgia	60
9	55–59	Female	Married	Yes	Yes	Daily	Junior high school	Shop assistant	>10 years	Knee OA	70
10	65–69	Male	Married	Yes	Yes	Daily	University	Teacher	>10 years	Bilateral Hip OA	58
11	65–69	Female	Married	Yes	Yes	Daily	University	Teacher	>10 years	Fibromyalgia, hand OA	67
12	70–74	Male	Married	Yes	Yes	Rarely	Junior high school	Retired	6–7 years	Bilateral Hip OA	46
13	65–69	Male	Married	Yes	Yes	Monthly	High school	Retired	>10 years	None	51

### The hip osteoarthritis continuum

**Fig. 1:**
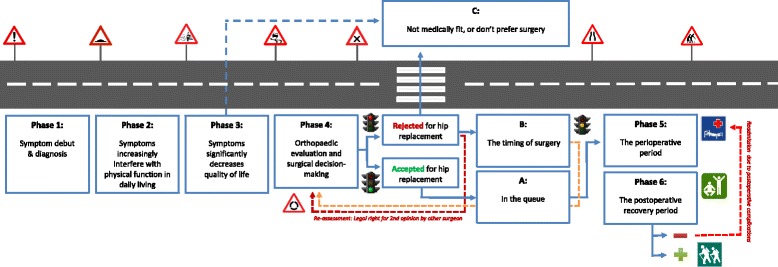
The hip OA continuum. Needs and help-seeking behaviour change over time dependent on disease severity, treatment response and decision-making processes

As a result of the qualitative analysis presented in the following sections and from reviewing the literature, we developed a care pathway model, *The hip OA continuum*, which illustrates a patients’ typical “hip journey” (Fig. 1). The model is divided into six phases, designed to demonstrate the informational and emotional needs of patients with hip OA, and how these needs may change over time related to the development of the disease, the clinical encounters within the health care services, and the subsequent decision-making processes. The patients' needs’ are analysed and condensed into key questions representing the main concerns of the patients at the respective phases along the continuum. Thus, this approach has a distinct person-centred focus.

1. The first phase represents the early hip OA-stage where the first symptoms emerge, diagnosis is set and an initial treatment plan is discussed.
2. The second phase represents the moderate hip OA-stage. This phase indicate a deterioration of symptoms, leading the patient to seek and try out more treatment options that might help the situation.
3. The third phase represents the severe hip OA-stage, where the symptoms are perceived as severe and more or less refractory to conservative treatment, leading up to a need of referral for orthopaedic evaluation.
4. The fourth phase represents the surgical decision-making phase with three possible options where the patient and the orthopaedic surgeon discuss whether or not the patient will benefit from hip replacement surgery. A) Patients who are accepted for surgery are placed in the waiting queue. These patients are advised to undergo pre-surgical muscle strengthening exercises and prepare for the postoperative recovery period at home, by procuring necessary ADL-aids. B) Patients who are rejected or choose to await surgery, are advised to continue conservative treatment until a renewed orthopaedic evaluation is warranted. If patients are unhappy about the final decision, they have a legal right to a second evaluation by another surgeon. C) This route represents an alternative route for patients who are not medically suitable for, or for some reason do not prefer to undergo surgery. This route might be taken at any point through the continuum, and it includes all treatment recommendations, in addition to surgery as a final option. These patients need to come to an acceptance of the situation, while optimizing the available non-surgical treatment options in order to live a meaningful life despite pain and its accompanying limitations.
5. The fifth phase represents the perioperative phase when patients follow a standardized hip-replacement programme. The patients receive interdisciplinary pre- and postoperative care, guided instructions and training concerning movement restrictions and general take-home physical exercises that stimulate optimal joint function.
6. The sixth phase represents the postoperative recovery period. The minority of patients who at some point experience post-operative complications may be re-admitted to the orthopaedic clinic for revision surgery, whereas the majority can enjoy a functional hip, although requiring a substantial rehabilitation period until fully recovered.

It is important to point out that for some patients this hip-journey may last for several years, but only months for others, reflecting individual differences in patients’ disease development, health seeking behaviour and access to appropriate health care services.

### Patients´ key questions through the hip OA continuum

An overview of the patients’ questions along with a selected empirical example are given in Table 2. A thick description of the participants' experiences follows, structured under each phase of the continuum. We include a comprehensive presentation of verbatim quotations in order to give the participants a voice and to display more of the empirical grounding for the six-phased model and the condensation of meaning into key questions. At the end of the description of each phase, the patients’ experiences are summarized.

**Table 2:** Phases and questions throughout the hip-OA continuum

Phase	Key questions	Typical quotes
1. Symptom debut & diagnosis	Something is wrong, what is this hip pain?	I had noticed this pain in my hips that persisted over a period of time. Then I told my GP about this pain, and he referred me for an X-ray (P3).
2. Symptoms increasingly interfere with physical functioning	My hip really bothers me, what can I do?	I haven’t taken any painkillers. I don’t want to […] I believe that if you take painkillers you’ll become worse and get more pain in the end (P5)
3. Symptoms significantly decreases quality of life	I can´t stand the pain, is it time for surgery?	I have to crawl up the stairs using the arms to push myself upwards (P8)
4. Orthopaedic evaluation and surgical decision-making	Will a hip replacement help me with my problems?	I have long been aware that I would need to replace the hip at some point, but I wanted to wait as long as possible (P3).
4a. The timing of surgery	The doctor tells me that the timing is not right for me, what now?	The doctor said that it (the joint) was worn out, but not enough to allow surgery. Then I just had to wait until it was bad enough (P6).
4b. In the queue	I am waiting for surgery, what should I do?	I have done exercises three times a week the last 3–4 months to prepare for the operation. It is important to strengthen the muscles to become best prepared for the period after surgery (P4)
4c. Not medically fit, or don’t prefer surgery	What are my options if I am not receiving surgery?	I have come to a point to wonder whether surgery is a wise thing to do - I’m not so happy about that either you know (P9)
5. The perioperative period	What will happen at the hospital?	They were very good at informing me about what to expect during the hospital stay- it was excellent information! (P10).
6. The recovery period	What can I expect after having surgery?	I was told that the prosthesis isn’t worth anything without the muscles- it must be rebuilt… I therefore chose to take part of all available training at the rehab centre (P12).

### Phase 1: The early stage of hip OA - symptom debut and diagnosis

In the early hip OA stage, some participants experienced the hip pain as vague or generalized, characterized by intermittent pain and stiffness. Many participants also described concurrent pain experiences in other joints than the hip. Depending on the participants’ health care seeking behaviour and the perceived severity of pain and its interference with daily functioning, they visited their GP to get an explanation of what it was.

*About 10 years ago, I noticed something wrong with my ankle, and then it spread upwards… I then started to get problems with my hip, when I sat down – when I got up and things like that… It felt like something was creaking in there, and I surely hoped it was muscular. I don’t remember how long it is since I had my first X-ray, but I was permitted one, and they confirmed wear in the joint.... it wasn’t that bad in the beginning, but pretty soon afterwards, it took a turn for the worse (P10, male 65).*

Some participants clearly had waited some time before seeking help from their GP, demonstrating individual differences in health care seeking behaviour.

*I had hip pain for a longer period of time. I mentioned it to my GP that I had a lot of pain in my hip. I would describe it as intermittent, but with some constant levels of pain. And then I was referred to… yes, for an X-ray maybe? I can’t remember if it was an X-ray or MRI, but nevertheless– one evening the GP called me at home and said that it was not unusual that I had this pain, because the hip joint was totally worn out… and that is quite a few years ago, at least seven years (P3, male 60).*

Some participants had a tendency to trivialize their experiences or play down their concerns by referring to possible natural causes, like getting older, pain conditions running in the family, or by comparing their problems with others.

*I think that as you get older, you expect such things to occur… things you might have to accept […] I have to admit that now, as a 65 year old – my father was 63 when he died – I think that I am so lucky to still being able to work and be active, and you see so many others that are incapable of that… you know? They suffer from far more serous conditions – everything is worse than this… It’s not serious in a way – nothing really to talk about (P11, female 65)…*

Some also indicated a feeling of shame attached with talking about their emergent hip pain or OA diagnosis, and felt that their hip disease was lower in the hierarchy than other diagnoses, such as cancer and diabetes type 2.

*It’s like…… almost a bit embarrassing to talk about…… it’s something that you kind of need to expect when you are so lucky to get old… […] I believe there is a hierarchy – a status related to different diseases among.... in the health care system, yeah? When I got cancer, I was sort of at the top of the pyramid (P11, female 65).*

Some participants clearly stated that they did not exclusively visit their GP to discuss their hip problems, but that it was brought up as an implicit concern at the end of the consultation.

*I have diabetes and I have typically discussed my hip problems when I have been to my doctor to check my blood glucose (P2, female 84).*

A common finding across the data was that the participants had not received general information about OA and pain management from their GP. Most of the participants did not actively seek information during consultations. This was explained partly by the fact that they did not know what to ask specifically and because the GP was not perceived to have the necessary expertise about OA.

*GPs aren’t specialists in osteoarthritis, so they.... they do what they can to refer me within the health care system, so I can’t say that I have anything to complain about in that respect… I get help for things I ask about, but I don’t really expect to receive any particular kind of information (P9, female 59).*

One participant explained that she had chosen to replace her GP.

*I decided to replace this GP immediately, because I thought… I was a bit mad at him - I had lived and struggled with this for so many years without anything being done. They must dedicate more time to each patient, right? (P1, female 88).*

One participant expressed great satisfaction with her GP, however, demonstrating the value of having an attentive GP.

*I can tell you that I have the world’s best GP! She is 67 years old and dreads that she must retire at 70. She is so strict! She gives me all the information I need and instructs me what to do. - You should do this kind of exercises and you’ll have an X-ray and you should do this – she arranges the whole show. Everything comes into my mailbox and I check it regularly (P4, female 70).*

Some found it difficult to appropriately disclose their concerns to their GP.

*You know, when you visit the doctor you do it because of the pain… but .you don’t know what causes it.... it’s therefore a bit difficult to know what to ask about (P9, female 59).*

Many discussed available time as a barrier.

*The GP is just keen to get you out in order to let the next patient in… He listens to what you say, but haven’t taken it seriously enough (P3, male 60).*

The common experience patients have at this initial stage may be summarized in the question: “Something is wrong, what is this hip pain?” The patients’ experiences an unfamiliar hip-related pain sensation that may require a visit to the GP. The GP acquires the medical history, performs relevant examinations (e.g. Harris hip score) and refers the patient to X-ray examination to set the diagnosis OA of the hip. The patient receives basic and variable information about OA and available treatment options depending on the severity of the symptoms and the GP’s and other health professional’s competence and communication skills.

### Phase 2: The moderate pain stage of hip OA – symptom deterioration and decreased physical function

As the hip problems evolved, the participants experienced increased pain, causing difficulties in performing regular and self-care activities.

*When I have taken a shower, I have troubles drying my feet. I can’t reach down, and I can hardly manage to put my socks on. I can’t care for my toenails - cutting them for example - that is really difficult on this leg. Then I have to wear long trousers - grab the trouser leg and force the left leg on top of the right one. When I do that I get a lot of pain.... I somehow manage to complete the task, but it’s no solution to continuously cross the pain threshold – I have done that increasingly often lately (P4, female 70)…*

Most of the participants had several x-rays, hoping that it would provide some answers.

*When results of the X-ray came, the radiologist had told the doctor that nothing was wrong with me – they didn’t find anything.... Ok, it thought, then there was nothing I could do about it… But with time it became worse, and I was....... I managed to visit the doctor a few times, and eventually they advised me to try those needles (acupuncture)....... (P1, female 88).*

Most participants had clear objections about taking pain killers on a daily basis, although the doctor had prescribed it. They felt there were too many pills to consume with potential troublesome side effects. A recurrent description was not being comfortable with taking pills in general, with statements such as “I’m not a pill-person”.

*It’s not ok to carry on like that....... But they....... they just prescribe pills- and I’m not a pill person....... I said to them that pills only relieve the pain, it doesn’t....... you’re hip will only get worse, and it doesn’t cure you....... You know, when you have taken pills over a period of time you’ll eventually need even stronger ones (P1, female 88).*

One participant explained that he did not take painkillers because he believed that it might make the situation worse in the long run.

*No, I don’t believe in that. I think that if you take pain killers, it will eventually lead to even more pain and worsening of the joints....... you won’t feel the pain and then the situation gets worse - I believe so anyway....... I would rather choose to rest – when you have had some rest, the pain isn’t so bad (P5, male 75).*

Despite the general picture of non-adherence to pain medication, some felt that taking painkillers on a regular basis helped them when they tried to cope with the situation.

*The point is to try to manage it as long as possible. I have taken....... what have helped me so far is that I have taken a slow release tablet- one Ketoprofen every morning for breakfast....... By doing that I actually have managed to live with the pain. That is in fact the reason why I have been able to cope for so long (P3, male 60).*

Some participants described alternative strategies to cope with the pain.

*I try to concentrate on other things than sickness and pain....... Yeah- simply re-focus. That my focus should be on other things, and I also try to stay positive! (P4, female 70).*

Most of the participants in this study did not attend physiotherapy on a regular basis. Some explained that they felt it was unnecessary because they had other ways of staying physically active.

*I don’t think there is any point in attending physiotherapy ....... I don’t think they make a difference. The one I had didn’t anyway, and I felt kind of cheated. The only thing I did was ride a bike, that’s all (P5, male 75).*

Experiences attributed to the moderate hip OA stage can be summarized in the question: *My hip really bothers me, what can I do?* The patients experience that the hip problems continue to evolve, causing decreased physical function due to increased and more persistent pain levels throughout the day. They have various ways of adapting and coping, and some try alternative treatment options. Most continue to live as before, but using activity modification as a strategy to cope with pain and avoid pain exacerbation. Patients become more dependent on help from others for physically challenging household chores. Most have tried physiotherapy, but with various results. Some take pain medication in order to stay active, but most do not because of misconceptions or fears of possible side effects.

### Phase 3: The severe pain stage of hip OA– the emerging need for an orthopaedic evaluation

When the symptoms significantly restricted abilities to do desired activities in daily living, some participants expressed that they had explicitly requested referral to an orthopaedic surgeon.

*It is very unpleasant. I’m pretty active these days - have a lot to do and I want to make the most out of my days. So, it bothers me a lot....... that is why I went to the doctor and said that we need to fix my hip. I can’t live with this – its impossible (P5, male 75).*

A decision for surgery may be driven by the hope for a better life.

*When I hear and see that people get a significantly improved life after surgery....... then, yes....... that makes me think about it as well. I have independently come to the conclusion that if it can help me to live a better life, I’m opting for surgery (P8, female 65).......*

Work responsibilities can influence a decision or desire to undergo hip replacement.

*I had plans to do this last year, but I received a job offer that......... I have to do this kind of work during the winter season, so that I don’t make things worse for myself, huh? So, it’s about planning… And then, yet another year has passed. If so much time elapses, then another year goes by. That is how I have thought about it for many years really (P3, male 60).*

One participant wanted surgery in order to be able to work, but was advised to wait.

*What has annoyed me the most is that they won’t do a joint replacement… If only for the knee, so that I might perform better at work– during the time I have left… They say that as long as I manage as it is now, it’s not worth doing surgery, due to the fact that they can’t give any guarantees that it will turn out for the best (P9, female 59).*

Most participants explained that their social network of friends, neighbours and family was an important source for information and advice relating to decision-making about total hip replacement.

*I have a pretty wide social network, and many of them have in fact undergone hip replacement. That makes us talk about these things. It becomes a topic for discussion, and we give each other advice (P4, female 70).*

Learning from others’ experiences provided hope for a better future.

*When you have suffered long enough you’ll try almost anything… learning from the experiences of others I have talked to, who have had a successful hip replacement– it makes me see possibilities for myself as well (P13, male 65).*

Undergoing hip replacement was perceived as a common procedure with excellent outcomes.

*You know, everyone has heard about someone who has replaced hips, and with such good outcomes– the surgeons now have such effective techniques with excellent outcomes (P3, male 60).*

Topics concerning possible risks for pre- and postoperative complications seemed to be of little importance in deliberation about undergoing THR. One stated that there are no guarantees in life.

*He said (the orthopaedic surgeon) that you never know how it turns out… But I’m ready to take that chance– no one have absolute guarantees for anything in life, yeah? (P1, female 88).*

Others relied heavily on the experience and competence of the orthopaedic surgeon.

*I consider the probability of that occurring very small, because I have always had confidence in specialists, and I trust them to deal with it… I won’t enter the operating theatre being scared! (P13, male 65)*

One participant brought up personal experiences with surgery performed earlier as grounds for deliberation about possible risks.

*Of course there is a chance, but with the two other operations that I’ve had, the gall bladder in 1999 and this (other hip) last year– it went just fine! I didn’t experience any problems with those… (P6, female 73)*

The common experience patients have at the advanced stage may lead up to the question: *“I can’t stand the pain, is it time for surgery?”.* As the disease progresses and interferes with the patient’s physical function and well-being, the GP prescribes pain medication, refers the patients to a physiotherapist and gives some advice on how the patient should adapt and self-manage. While some patients find satisfactory ways to cope at this stage, some do not respond effectively and experience that the disease continues to progress, leading to significantly decreased physical function and quality of life. The pain levels increase and the sleep quality decreases as a consequence of frequent pain incidents during the night. These patients may be considered for a specialist evaluation to decide whether hip replacement is appropriate.

### Phase 4: Orthopaedic evaluation and surgical decision-making

Participants stated that pain was the main reason for considering hip replacement surgery.

*The reason was to get help and pain relief. It is… it’s just that. Yes, I don’t exactly know why I asked to be referred, but I have had such terrible pain and currently have terrible pain. I kind of consider my future prospects as dark if I have to live like this the rest of my time! If anything can be done… but I don’t know, maybe I can do more– exercise more, or?… (P8, female 65).*

A common finding was that it seemed important for the participants to discuss experiences with others who had undergone hip replacement.

*It’s something you really need to embrace… it’s a mental… you kind of surrender one part of the body… voluntarily, you know? I therefore found it helpful to listen to their story about the time before and after surgery. I found it very important to learn from their experiences… (P10, male 65).*

Participants who were denied hip replacement were told by their GP or orthopaedic surgeon that the features of the X-ray did not show significant changes that would allow for a hip replacement, or they were categorized as too young and therefore advised to postpone the procedure as long as possible.

*The X-ray showed very little radiographic changes, and they kind of laughed at me and… I asked them if any help is available for this… No! Come and see us in three years they said – that was it… (P8, female 65).*

This phase represents the process of surgical decision-making and can be summarized by the question: “Will a hip replacement help me with my problems?”. This illustrates a complex process that is regarded as “the point of no return”, as one participant explained. After having updated hip x-rays, the orthopaedic surgeon and the patient review the current situation together. Many participants said that reaching this phase was a relief - something they knew existed as a final option and that might help the situation significantly. Decision-making for THR seems to start before the actual meeting with the orthopaedic surgeon, either as a result of advice from health professionals or after observing others’ experiences from undergoing THR. The patients know that this is a final and effective option, but the issue of timing depends on several factors. The question “The doctor tells me that the timing is not right for me, what now?” illustrates rejection as a possible outcome of surgical decision-making. In Norway, these patients have a legal right to a renewed evaluation and may opt for this if they disagree with the decision. Otherwise, these patients continue at their best with conservative treatment, but may be referred for a second orthopaedic evaluation at a later point in time. The question “I am waiting for surgery, what should I do?” reflects the outcome where patients are accepted for hip replacement and placed on the waiting list.

### Phase 5: Perioperative period

Most participants who were due for THR had been advised to do joint muscle-strengthening exercises prior to hospital admission.

*I was told that it is smart to strengthen the muscles… I therefore started intensive training, but it was far too late… I started 14 days prior to surgery with intensive training, but I realize that I should have started much earlier. Clearly! (P10, male 65).*

However, one participant had been informed that muscle strengthening was contraindicated in the pre-operative period.

*We were told that there was no use in doing that… It was no benefits doing surgery on hard muscles… The soft muscles however, the ones that were little trained was better –– they healed more quickly and you would have a shorter recovery period (P12, male 72).*

Elective patients receive standardized information prior to admission that explains what to expect and what they should do to prepare for the recovery period.

*I think that the information I received here at the hospital – the letters about where to meet, when and what tests to take – generally, the whole course of the operation. I think it was fantastic! (P4, female 70).*

Most of the participants in this study expressed satisfaction about the information provided by the hospital staff.

*The health providers here are very informative and good at telling what to do and what to expect. It makes you gain confidence in the staff… Yeah, I think it has been very satisfying! (P5, male 75).*

After hospital admission, patients follow a standardized treatment and rehabilitation program. Most patients are discharged during the first or second postoperative day, but with an expectancy of a 6–12 month recovery period to regain full physical strength and energy. This phase is reflected by the question: “What will happen at the hospital?”. It represents a range of possible concerns and expectations that the patients might have during this period of the continuum. They are in need of professional guidance on how to adapt to a life with a prosthesis, and they learn ways to optimize its function and minimize the likelihood of complications.

### Phase six: Postoperative recovery

Patients are normally not offered in-house rehabilitation following THR in Norway. Most of the participants in Group One who had surgery the next day expected to return to their homes after discharge from the hospital. However, one of participants in Group Two who had previously undergone THR explained the value of admission to a rehabilitation unit.

*I spent four weeks, that is the maximum time allowed… there you can get all the training you need. Cycling, gymnastics of all kinds, training with weights on your feet, and climbing up and down the steps on a ladder… yes– overall, to get into shape… I don’t question that this is what has helped me improve this much (P12, male 72).*

The question; “what can I expect after having surgery?” reflects the needs of patients who have undergone hip replacement. They are pre-informed before surgery and have completed preparations for this period, but are in need of support and information about ways to prevent postoperative complications. All patients are expected to stay physically active in order to optimize stability and function in the hip joint.

### The alternative route: The non-surgery option

Patients who are not considered medically appropriate for surgery, or for some reason do not prefer the procedure, seem to have a need to accept the situation, while optimizing the available non-surgical treatment options in order to live a meaningful life despite pain and its accompanying limitations. One of the participants had ambivalent thoughts regarding surgery.

*I honestly don’t know… you hear what I’m saying – I have come to a point to wonder whether surgery is a wise thing to do. I’m not so happy about that either, you know (P9, female 59).*

One participant said that it was essential to accept the situation, but this did not mean one should let go of the hope that help could be obtained.

*I do feel that I accept it, I really do… I have to! I must accept it, but I am still thinking about whether there might be something that can help. I always do (P8, female 65).*

This alternative route is reflected through the question: “*What are my options if I'm not receiving surgery?”.* This route is included as a possible outcome from surgical decision-making, as some patients may be ineligible for THR and others are cautioned because they have a greater than average risk of post-surgical complications (i.e. Osteoporosis, obesity, diabetes mellitus or heart disease). Some patients may also choose not to receive surgery due to personal reasons (i.e. concerns, previous experiences with surgery, advice from others).

## Discussion

The qualitative approach used in this study revealed findings that capture several aspects of the complex nature of how hip OA patients experience the development of their illness, including the encounters with the health care system and the clinical decision-making processes. The model presented with the accompanying key questions give an overview of the OA hip-journey, based on the participants’ experiences and research literature. We believe that it provides a simplified, but realistic map of how patients with hip OA navigate and interact with health professionals throughout the continuum. The identified questions are condensed representations of a range of related questions that patients have across the continuum. These questions need to be appropriately addressed in order to increase the patients’ possibilities for effective self-management and informed decision-making.

### Patient needs

The majority of the participants did not seem to receive information relevant to self-management and surgical decision-making, but at the same time they didn’t expect to be given this kind of information from their GP. Most of the participants did not adhere to a prescribed pain medication plan. Furthermore, most did not experience benefits from individual training with a physiotherapist. As a result, they struggle to find effective pain-management strategies in daily living, often including avoidance of activity resulting in increased pain and decline in physical function. Similar and other barriers to utilization of recommended treatments have been reported in several studies [12, 15, 39, 40]. Sanders et al. [41] identified barriers related to three stages: first, some were reluctant to present themselves for treatment because of their perceptions that arthritis was part of normal ageing and that there was little that could be offered to them; second, while many had consulted GPs, their experiences were mostly negative, with GPs appearing to confirm the lack of effective treatment and rarely offering referral to secondary care; and third, waiting lists and rationing were perceived to be a barrier to getting treatment in secondary care and sometimes surgery appeared to be denied because they were considered ‘too young’ or not sufficiently disabled.

These barriers may partly be explained by a gap in essential knowledge about OA and available treatment alternatives [16, 42, 43]. Dissemination of key information about OA is one approach that could increase patients’ knowledge and abilities to make informed decisions. Key messages are the important information a patient needs to know about a disease, its causes, its diagnosis, and its management. A total of 21 key messages have been identified and prioritised through a multistage consensus process involving both OA experts and patients [44].

### Decision-making for total hip replacement

The general picture in this study is that patients are not appropriately informed about possible complications associated with THR. This is problematic. Although THR have low complication rates, patients should be realistically informed about possible pre- and postoperative complications. Only a few of the participants expressed explicit concerns about undergoing surgery, and none stated that they had discussed the associated risks with an orthopaedic surgeon or GP. It seemed that they generally put their trust in the orthopaedic surgeons’ professional skills and that risks, according to them, hadn’t been appropriately addressed when they decided to undergo THR. When asked about the type of information that was perceived as important during orthopaedic consultations, most male participants explained a need to understand how the prosthesis is designed, and that the orthopaedist had drawn pictures and used models to demonstrate how it is fitted into the hip joint. Other aspects were related to an expectation of pain relief and restored physical function, and the abilities to return to work. These expectations have been reported as the most important pre-operative expectations that influence a decision for undergoing surgery [45]. Although few of the patients discussed concerns explicitly during the interviews, it is appropriate to assume that the setting (at the hospital) and timing of the interview (one day prior to surgery) might have affected their desires to talk about concerns that might cause additional anxiety or feelings of uncertainty. Nevertheless, studies demonstrate that patients make decisions for THR without raising all their concerns during clinical consultations. A study of 59 patients with hip or knee OA considering surgery identified a total of 164 expressed concerns by using telephone interviews, but found that the patients raised only half their concerns during planned audiotaped orthopaedic consultations. The identified concerns pertained to the surgery (anticipated quality of life after the surgery, the care facility, the timing of the operation, and the patient’s capacity to meet the demands of the surgery) and the surgeons (their competency, communication, and professional practices). Patients were highly selective about the concerns they raised; concerns about logistical aspects of surgery (about the care facility and the timing of the operation) and the anticipated quality of life after the surgery were often expressed, while concerns about their capacity to meet the pre- and postoperative demands of surgery were raised less often, and concerns about surgeons were rarely raised [46].

The process of decision-making for THR should ideally be initiated by the GP during the early phases of the continuum in order to provide time and opportunities for careful deliberation and to identify the optimal timing for referral to an orthopaedic surgeon. The patients need to establish their goals and expectations, examine their values and preferences and how they relate to their ability to cope with their condition, their need for pain relief, quality of life restoration, and the goals and expectations of having THR or not. They might need to acknowledge fears surrounding the procedure and any previous experiences. Physician opinion, the social impact of others, and knowledge and concerns about recovery are also factors that may impact patients’ decisions regarding surgery [47]. Shared decision-making (SDM) is increasingly advocated as an ideal model of treatment decision-making in the medical encounter [48]. This includes an evolution from the paternalistic model, in which the physician tells the patient what the treatment will be, to a more patient-centred approach [49]. The SDM model allows both the physician and the patient to actively contribute towards the medical decision. Physicians educate the patient about the treatment options available for their condition, whereas patients indicate how their preferences and values relate to these options. The patient and physician then work together in order to reach a consensus in a two-way exchange of information that enhances the potential outcome for the patient [47].

### Strengths of the study

We were able to recruit a relatively broad sample of participants in terms of age, gender and distribution within the hip OA continuum. By conducting in-depth interviews, we produced a rich source of qualitative data which contributes to the understanding of how patients might experience living and coping with hip OA within the Norwegian health care system. It is one of few studies that focuses on the whole hip OA continuum of care, as seen from a person-centred perspective. The proposed model and questions can act as a guide to help patients and their families to visualise the complexity of the disease, what to expect and their role as active participants in communication and decision-making.

### Limitations

In this study we have made an effort to illustrate a typical hip OA continuum and further describe accounts that represent patients’ informational and emotional needs expressed explicitly or implicitly by the participants. It is clear that all patients entering this OA continuum are heterogeneous human beings with individual needs that are impossible to fully account for through methods used in this study. A relatively small sample of patients were included, thus we expect that other important aspects related to patients’ information and emotional needs are missing from this presentation. A majority of the patients included in the study had developed severe hip OA, and were due for or contemplated surgery. It is therefore likely that patients at early stages of hip OA are underrepresented in the sample and their experiences and questions less clearly explored. A future study could use the hip OA model as basis to recruit and allocate participants to each phase, and to assess specific needs expressed by both patients and their care providers.

## Conclusions

This study reveals that patients with hip OA are in great need of information and emotional support throughout the whole hip OA continuum. Patients’ needs, concerns and expectations must be appropriately understood and met by health providers caring for hip OA patients. The knowledge and model developed through this study will be used as a basis in the development of a web-based tool for hip OA patients, their families and involved health providers designed to increase patients’ knowledge and support communication during the process of preparing individualized care plans in clinical settings.
